# Carriage and antimicrobial susceptibility of commensal *Neisseria* species from the human oropharynx

**DOI:** 10.1038/s41598-024-75130-9

**Published:** 2024-10-23

**Authors:** Victoria F. Miari, Wesley Bonnin, Imogen K. G. Smith, Megan F. Horney, Samer J. Saint-Geris, Richard A. Stabler

**Affiliations:** 1 Department of Infection Biology, Faculty of Infectious and Tropical Diseases, London School of Hygiene & Tropical Medicine, Keppel Street, London, UK; 2 Department of Microbiology, Whittington Health NHS Trust, London, UK

**Keywords:** Commensal, *Neisseria*, Antimicrobial resistance, Whole genome sequencing, Antimicrobial resistance, Next-generation sequencing

## Abstract

Commensal *Neisseria* (*N*c) mainly occupy the oropharynx of humans and animals. These organisms do not typically cause disease; however, they can act as a reservoir for antimicrobial resistance genes that can be acquired by pathogenic *Neisseria species*. This study characterised the carriage and antimicrobial susceptibility profiles of *N*c from the oropharynx of 50 participants. Carriage prevalence of *N*c species was 86% with 66% of participants colonised with more than one isolate. Isolates were identified by MALDI-ToF and the most common species was *N. subflava* (61.4%). Minimum inhibitory concentrations (MICs) to penicillin, ceftriaxone, ciprofloxacin, azithromycin, tetracycline, and gentamicin were determined by agar dilution and E-test was used for cefixime. Using *Ng* CLSI/EUCAST guidelines, *Nc* resistance rates were above the WHO threshold of 5% resistance in circulating strains for changing the first line treatment empirical antimicrobial: 5% (CLSI) and 13 (EUCAST) for ceftriaxone and 29.3% for azithromycin. Whole genome sequencing of 30 *N*c isolates was performed, which identified AMR genes to macrolides and tetracycline. Core gene MLST clustered *Nc* into three main groups. Gonococcal DNA uptake sequences were identified in two *Nc* clusters. This suggests that *N*c have the potential AMR gene pool and transfer sequences that can result in resistance transfer to pathogenic *Neisseria* within the nasopharyngeal niche.

**Supplementary Information:**

The online version contains supplementary material available at 10.1038/s41598-024-75130-9.

## Introduction

*Neisseria* species are gram-negative aerobic cocci, part of the β-proteobacteria class. *Neisseria* colonise the mucosal surfaces of humans and animals, mainly the oral cavity and nasopharynx. To date, there are at least 43 published *Neisseria* species by the List of Prokaryotic names with Standing in Nomenclature (LPSN; accessed 4 May 2024)^1^ and 47 by the National Center for Biotechnology Information (NCBI; accessed 4 May 2024)^2^. The predicted phylogeny of *Neisseria* species is continuously evolving. Studies performed using 16s rRNA sequencing and conserved housekeeping genes identified five separate groups of *Neisseria*^3^. Group one contained *Neisseria gonorrhoeae (Ng)*, *Neisseria meningitidis (Nm)*, *N. polysaccharea*, and *N. lactamica*, group two included *N. subflava*, *N. flavescens*, and *N. mucosa*. The third group included only *N. cinerea* strains. The fourth and fifth groups contained *N. phayngis* and *N. elongata* species respectively^3^. More recent studies however have suggested the re-classification of certain species into single clusters, for example *N. perflava*,* N. subflava* and *N. flava* are now thought to belong to the *N. flavescens* group^4^. Genomic relatedness among *Neisseria* species has been examined by several methods, but core genome MLST (cgMLST) is now commonly used^5,6^.

The ability of *Neisseria* species to uptake DNA and integrate it into their genome is a common feature among the genus leading to a high degree of genetic variation, which is crucial to survival and adaption to their host^7^. Uptake of DNA in *Ng* is regulated by the presence of the 10-base pair DNA uptake sequence (DUS) 5’-GCCGTCTGAA-3’^8^. More recently, a revised 12-base pair sequence was identified (AT-DUS: 5’-AT-GCCGTCTGAA-3’), which enhances transformation efficiency^9^. A variant DUS (vDUS 5’-GTCGTCTGAA-3’) present in commensal *Neisseria* (*N*c) has also been described, with some species such as *N. mucosa* having > 3,000 copies^10^.

Commensal *Neisseria* are important reservoirs of transferable antimicrobial resistance (AMR) for pathogenic species^11,12^. The transfer of β-lactam resistance, including extended spectrum cephalosporins (ESC) is of particular importance; *Nm* and *Ng* strains resistant to β-lactams have been shown to harbour mosaic *penA* genes, acquired from *N*c species such as *N. cinerea* and *N. perflava*^13,14^. As such, it has been suggested that surveillance of *N*c species can contribute to delaying the spread in AMR in pathogenic *Neisseria* species^15^.

The prevalence of *N*c in the oropharynx and associated AMR is understudied compared to pathogenic *Neisseria* species. However, *N*c prevalence has been estimated between 10.2% and 100%^16–20^, with some studies reporting individuals’ colonisation by up to four different species^17,18,20^. Susceptibility of *N*c to ceftriaxone is low, with reported median minimum inhibitory concentrations (MICs) of 0.047 mg/L^21^, 0.002 mg/L^22^ and 0.03 mg/L^23^, although the last two studies were limited to only *N. lactamica* and *N. subflava* respectively. Additionally, resistance rates to ceftriaxone and cefixime among *Nc* has been estimated as 28% and 31% respectively^17^.

To our knowledge, this is the first study to report *Nc* prevalence combined with penicillin, ceftriaxone, ciprofloxacin, azithromycin, tetracycline, and gentamicin MICs and genomic analyses, and the only one to date performed in the United Kingdom. This study highlights that *N*c have the potential AMR gene pool and transfer sequences that can result in resistance transfer to *Ng* and *Nm* within the nasopharyngeal niche.

## Methods

### Participant recruitment and sample processing

A cross-sectional study of staff and students from the London School of Hygiene & Tropical Medicine (LSHTM) was undertaken between June and July 2019. Any participant over the age of 17 years old was eligible for inclusion, with the following exclusion criteria: antibiotic use within one month, usage of antiseptic mouthwash in the past week and participants who are taking steroids or immunosuppressant therapy. The aims of the study were explained to all participants, after which informed consent was obtained. All subsequent experiments were performed in accordance with the relevant guidelines and regulations.

A DrySwab device (MWE, Nottingham, UK) was used to sample the peritonsillar areas of participants. Swabs were expressed in 1mL of sterile saline by vortexing vigorously, and 50 µL inoculated onto a Luria-Bertani Vancomycin Trimethoprim Sucrose Neutral Red (LBVT.SNR) agar, as previously described^18^. Briefly, LBVT.SNR agar consisted of 1% tryptone (Oxoid, Basingstoke, UK), 0.5% yeast extract (Oxoid), 0.5% sodium chloride (Sigma-Aldrich, St. Louis, Missouri, U.S.), 1.5% Bacteriological Agar Number 1 (Oxoid), 1% w/v sucrose (VWR International, Radnor, Pennsylvania, US), 3 mg/L trimethoprim (Sigma-Aldrich), 3 mg/L vancomycin (Sigma-Aldrich) and 0.3% neutral red indicator (Sigma-Aldrich). Inoculated plates were incubated at 5% CO_2_ at 37^o^C for 48 h.

### Bacterial identification

Cultured isolates were first observed for colonial morphology, including colour, texture, and size. Morphologically distinct colonies from the LBVT.SNR agar were sub-cultured on chocolate agar (Oxoid) for further identification and antimicrobial susceptibility testing (AST). Oxidase and gram staining were performed on colonies of interest; oxidase positive, gram-negative cocci were considered as presumptive *Neisseria species*. Isolates were stored in 20% glycerol brain heart infusion (BHI) broth (Oxoid) at -70^°^C until further testing.

Identification to species level was determined by Matrix-Assisted Laser Desorption/Ionisation – Time-of-Flight mass spectrometry (MALDI-ToF MS), using a Bruker MALDI Biotyper (Bruker Daltonics, Billerica, Massachusetts, US). Identification values of 2.0 or over were accepted, while values under 2.0 were repeated once.

### Antimicrobial susceptibility testing

Minimum inhibitory concentrations for penicillin, ceftriaxone, ciprofloxacin, azithromycin, tetracycline, and gentamicin (Sigma-Aldrich) were all determined by agar dilution in line with the Clinical and Laboratory Standard Institute protocol^24^, using gonococcal medium base (GCMB) agar (BD Difco, Franklin Lakes, New Jersey, US). Cefixime MICs were obtained by E-test (Biomerieux, Marcy-l’Étoile, France), on GCMB. Gonococcal WHO controls K, G, V, F, X and Y^25^ were included in the AST, due to the lack of *N*c control strains. Isolates with a penicillin MIC > 1 mg/L were tested for β-lactamase production using a cefinase disk (Oxoid), according to manufacturer’s instructions. As there are no MIC breakpoints for *N*c, calculated rates of reduced susceptibility (referred to as resistance for ease) used the Clinical & Laboratory Standards Institute (CLSI)^24^ and European Committee on Antimicrobial Susceptibility Testing (EUCAST v.13.1)^26^. Gentamicin breakpoints used epidemiological values suggested previously^27^ (Table 2). Resistance rates to all antimicrobials were calculated for all *N*c overall and for each species individually (Table 2).

The MIC values generated were used to deduplicate isolates within individual patients, using the following criteria:

1. Isolates with the same phenotypic appearance on LBVT.SNR agar, and.
2. Isolates with same species ID by MALDI-ToF, or whole genome sequencing (WGS) where MALDI did not give an ID, and.
3. Isolates with at least five out of seven antibiotic matching MICs, within 1 log_2_ MIC.

### Whole genome sequencing and bioinformatic analysis

Total genomic DNA was extracted using the PureLink Genomic DNA Mini Kit extraction kit (Invitrogen, Waltham, Massachusetts, US) and quantified using the Qubit dsDNA BR assay kit (Invitrogen). The Nextera XT library (2 × 151 bp) prep kit (Illumina, San Diego, California, US) was used to prepare the sequence libraries as per manufacturer’s protocol. The samples were sequenced on a MiSeq System (Illumina) as per the recommended protocol. Additional Illumina (2 × 251 bp) sequencing was performed at MicrobesNG (MicrobesNG, Birmingham, UK). Raw sequence data were quality controlled using Trimmomatic v0.38^29^ with the following specifications: Leading:3 Trailing:3 SlidingWindow:4:20 Minlen:36. Quality control (QC) checks were performed using FastQC v0.11.8^30^. Fastq reads were mapped against reference sequences using BWA MEM with default settings^31^ and viewed in Artemis and ACT^32,33^. *De novo* sequence assemblies were performed using Spades v3.13^34^ with default settings, a coverage cut-off of 20 and k-mer lengths of 21, 33, 55, 77, 99 and 111. Draft genome multi-fasta files were evaluated using Quast assessment tool v5.0.2^35^. Contigs were ordered against a *N. meningitidis* MC58 (accession AE002098) using ABACAS v1.3.1 using -dmbc settings^36^. Non-matching contigs were appended to the ordered contigs. The resulting assemblies were polished using Pilon v1.22 with default settings^37^ and annotation using Prokka v1.13 in gram negative mode^38^.

The assembled contigs were screened for AMR genes using ABRicate^39^ v1.0.1 and CARD^40^, and NCBI AMRFinderPlus^41^ databases and combined. Putative plasmid replicons were identified using the ABRicate with the PlasmidFinder database^42^. MLST profiles were determined using the software package MLST v2.16.1 from the draft assemblies^43^. Kraken2 using draft assemblies and the minikraken_8Gb_20200312 database^44^ was used to predict species. The BSR-Based Allele Calling Algorithm (chewBBACA)^45^ and predetermined *Neisseria* schema was used to generate cgMLST profiles and paralog removal using alleles present in 95%^46^. Allele profile data was used to generate a MSTree in Grapetree using --wgMLST and default settings^47^. Heatmaps was generated using Morpheus website (software.broadinstitute.org) with hierarchical clustering using Euclidean distance, average linkage method.

### Statistical analysis

All statistical analyses were performed with STATA 18 (StataCorp LLC, College Station, Texas, US). Prevalence and 95% confidence intervals (CI) were calculated for each of the *N*c species. The MICs between *N*c species was compared using the Kruskal-Wallis rank sum test. To enable statistical testing, MICs above the maximum or below the minimum range tested were converted to the dilution before or after the limit of detection, as previously described^21^. For example, azithromycin MIC > 256 mg/L was expressed as 512 mg/L.

## Results

### Participant demographics and ***Neisseria*** isolates

Fifty participants were recruited with 37 (74%) females and median age was 35 (range 17 to 81). The number of participants colonised with *N*c was 43/50, generating an estimated population prevalence of 86% (95% CI; 73.8%, 93%). In total, there were 143 morphologically distinct *N*c isolates cultured from the 43 participants. A total of 42 isolates were removed as duplicates, leading to a final total of 101 isolates from the 43 participants that grew *N*c.

### ***Neisseria*** species prevalence and characterisation

The most common *N*c species detected by MALDI-ToF was *N. subflav*a (62/101, 61.4%) (Supplementary Table S1). The second most prevalent species was *N. flavescens* (12 isolates, 11.9%), then *N. perflava* (10, 9.9%), *N. macacae* (6, 5.9%) and *N. mucosa* (3, 2.9%) (Supplementary Table S1). Twenty isolates (19.8%) were identified by MALDI-ToF as either one of two probable species, both having an index of over 2.0 (high confidence identification); the isolate with the highest index was considered as the primary ID (Supplementary Table S1). No ID was possible on eight isolates by MALDI-ToF; these were classified as *Neisseria spp* (Supplementary Table S1).

**Fig. 1:**
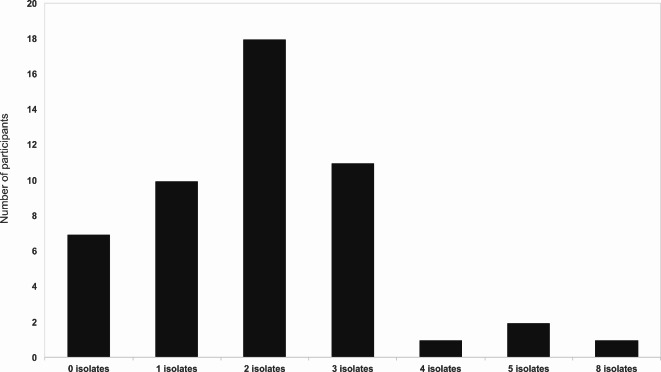
Number of *Neisseria* isolates present in the oropharynx of 50 study participants. Analysis was performed with results obtained from MALDI-ToF MS.

*N. subflava* had the highest incidence among the participants, with 74% (37/50 participants) carrying this species. This was followed by *N. flavescens* (20%, *n* = 10), *N. perflava* (18%, *n* = 9), *N. macacae* (10%, *n* = 5) and *N. mucosa* (6%, *n* = 3). Ten participants (20%) harboured a single *N*c species, however, some participants harboured multiple isolates; 18 (32%) participants were colonised by two isolates, 11 (22%) by three isolates, 2 (4%) by five isolates and 1 (2%) each were colonised by four and eight isolates (Fig. 1).

### Susceptibility of commensal ***Neisseria*** species

**Fig. 2:**
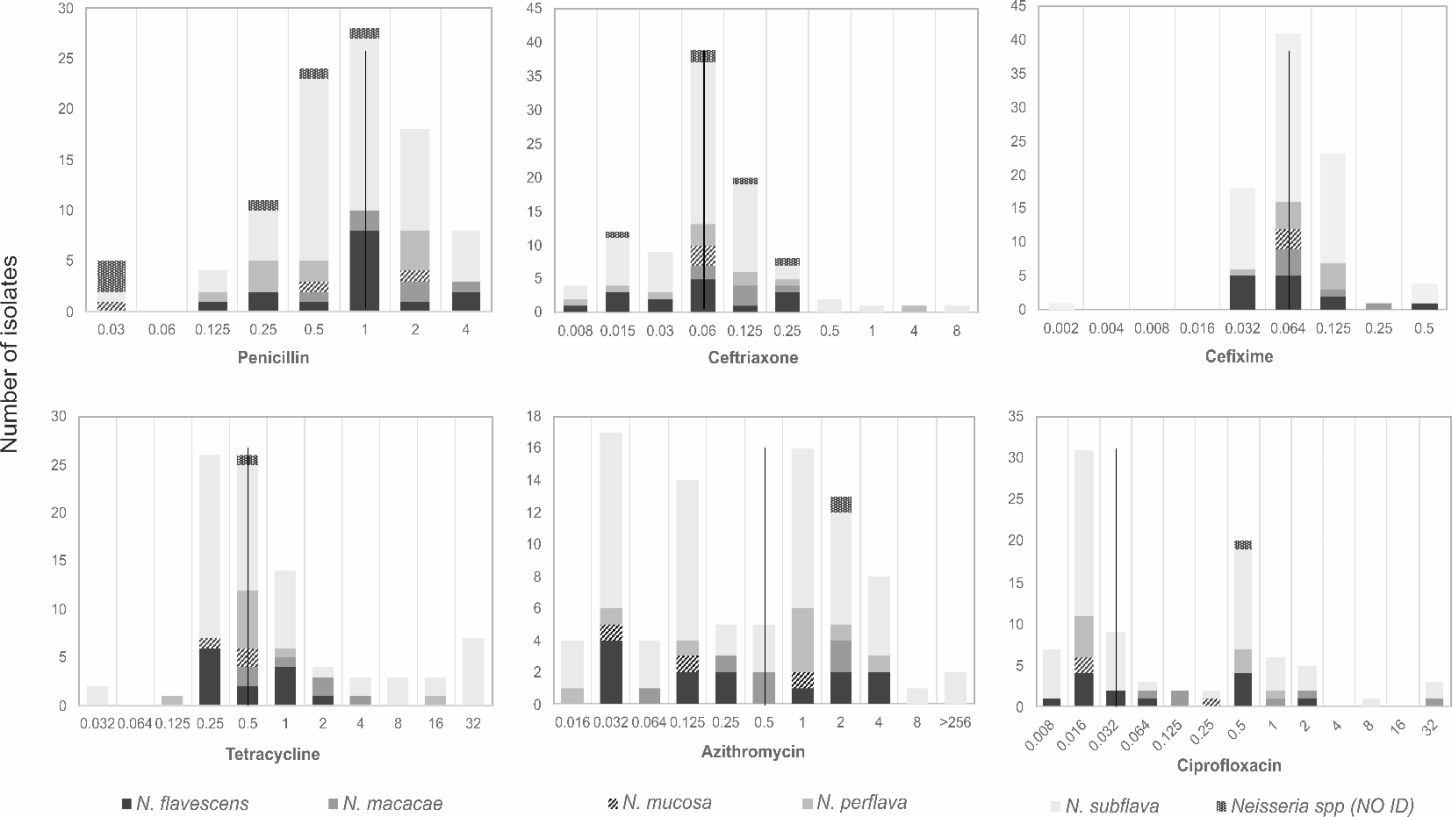
Minimum inhibitory concentration distribution of commensal *Neisseria* species to penicillin, ceftriaxone, cefixime, ciprofloxacin, azithromycin and tetracycline, performed by agar dilution. The dotted line indicates the median MIC for each antimicrobial.

After deduplication of isolates, the following MIC data were analysed: penicillin and ceftriaxone MICs for 101 and 100 isolates respectively and for cefixime, ciprofloxacin, azithromycin, gentamicin and tetracycline, 91 isolates MICs (Table 1). The median MICs for penicillin, ceftriaxone, cefixime, ciprofloxacin, tetracycline, azithromycin and gentamicin were 1 mg/L, 0.06 mg/L, 0.064 mg/L, 0.032 mg/L, 0.5 mg/L, 0.5 mg/L and 4 mg/L respectively (Table 1; Fig. 2 and Supplementary Table S2). No isolates produced a detectable β-lactamase. The proportion of isolates overall resistant to penicillin and azithromycin according to both CLSI and EUCAST breakpoints was 26.7% (27/101) and 29.3% (27/92) respectively (Supplementary Table S2). Of the penicillin resistant isolates, 10 were also resistant to azithromycin. *N. subflava* had the highest number of resistant isolates to both antibiotics (PEN; *n* = 15/59 [25.4%], AZI; *n* = 15/58 [25.9%]) (Table 2), with seven isolates being resistant to both antimicrobials. According to CLSI breakpoints, the proportion of isolates resistant to ceftriaxone, cefixime, ciprofloxacin and tetracycline were 5%, 4.3%, 16.3% and 22.8% respectively. The proportion of isolates resistant to these antibiotics differed by EUCAST breakpoints; they were 13.0%, 5.4%, 45.7% and 37%. No isolates were resistant to gentamicin.

**Table 1:** Summary of minimum inhibitory concentration characteristics by commensal *Neisseria* species and relationship between species and MIC.

Antimicrobial	PEN	CRO	CFX	CIP	TET	AZI	GEN
Median MICs (mg/L)							
*Neisseria* all *spp*	1	0.06	0.064	0.032	0.5	0.5	4
*N. flavescens*	1	0.06	0.047	0.032	0.5	0.25	4
*N. macacae*	1.5	0.125	0.064	0.5625	1.5	0.5	3
*N. mucosa*	0.5	0.06	0.047	0.016	0.5	0.125	4
*N. perflava*	0.5	0.07	0.064	0.016	0.5	1	4
*N. subflava*	1	0.06	0.023	0.032	0.5	0.375	4
*Neisseria spp (NO ID)*							
MIC^N^	101	100	92	92	92	92	92
Modal MIC	1	0.06	0.064	0.016	0.5	0.032	4
Range	0.03-4	0.015-8	0.002-0.5	0.008-32	0.032-32	0.016-512	0.5–16
IQR range	0.5-2	0.06–0.125	0.047–0.094	0.016-0.5	0.25-1	0.06–1.5	2–4
Geometric mean	0.7	0.07	0.06	0.09	0.81	0.37	3.47
Kruskall-Wallis^N^	90	88	86	86	86	86	86
H score	2.56	2.94	3	4.57	2.9	0.61	2.03
p	0.464	0.4	0.39	0.21	0.41	0.89	0.57

N, numbr of isolates; IQR, interquartile range; PEN, penicillin; CRO, ceftriaxone; CFX, cefixime; CIP, ciprofloxacin; TET, tetracycline; GEN, gentamicin.

**Table 2:** Resistance rates of commensal *Neisseria* species to the tested antimicrobials, interpreted by CLSI and EUCAST breakpoints for *Neisseria gonorrheae*.

		CLSI *N*^*R*^/*N*^T^ (%)						
		PEN	CRO	CFX	CIP	TET	AZI*	GEN^**§**^
Breakpoint (R >)		1	0.25	0.25	0.5	1	1	16
Overall		27/101 (26.7%)	5/100 (5%)	4/92 (4.3%)	15/92 (16.3%)	21/92 (22.8%)	27/92 (29.3%)	0/92 (0%)
*N. flavescens*		3/15 (20%)	0/15 (0.0%)	1/13 (7.7%)	1/13 (7.7%)	2/13 (15.4%)	5/13 (38.5%)	0/13 (0%)
*N. macacae*		3/6 (50%)	0/6 (0.0%)	0/6 (0.0%)	3/6 (50%)	3/6 (50%)	2/6 (33.3%)	0/6 (0%)
*N. mucosa*		1/3 (33.3%)	0/3 (0.0%)	0/3 (0.0%)	0/3 (0.0%)	0/3 (0.0%)	0/3 (0.0%)	0/3 (0%)
*N. perflava*		4/10 (40%)	1/10 (10%)	0/9 (0.0%)	1/9 (11.1%)	1/9 (11.1%)	2/9 (22.2%)	0/9 (0%)
*N. subflava*		15/59 (25.4%)	4/59 (6.8%)	3/58 (5.1%)	10/58 (17.2%)	15/58 (25.9%)	15/58 (25.9%)	0/59 (0%)
*Neisseria spp (NO ID)*		1/7 (14.2%)	0/7 (0.0%)	0/3 (0.0%)	0/3 (0.0%)	0/3 (0.0%)	3/3 (100%)	0/2 (0%)

		EUCAST N^R^/N^T^ (%)						
		PEN	CRO	CFX	CIP	TET	AZI*	GEN^**§**^
Breakpoint (R >)		1	0.125	0.125	0.06	0.5	1	16
Overall		27/101 (26.7%)	13/100 (13%)	5/92 (5.4%)	42/92 (45.7%)	34/92 (37%)	27/92 (29.3%)	0/92 (0%)
*N. flavescens*		3/15 (20%)	3/15 (20%)	1/13 (7.7%)	6/13 (46.2%)	5/13 (38.5%)	5/13 (38.5%)	0/13 (0%)
*N. macacae*		3/6 (50%)	1/6 (16.7%)	1/6 (16.7%)	6/6 (100%)	4/6 (66.7%)	2/6 (33.3%)	0/6 (0%)
*N. mucosa*		1/3 (33.3%)	0/3 (0.0%)	0/3 (0.0%)	1/3 (33.3%)	0/3 (0.0%)	0/3 (0.0%)	0/3 (0%)
*N. perflava*		4/10 (40%)	2/10 (20%)	0/9 (0.0%)	4/9 (44.4%)	2/9 (22.2%)	2/9 (22.2%)	0/9 (0%)
*N. subflava*		15/59 (25.4%)	6/59 (10.1%)	3/58 (5.1%)	24/58 (41.3%)	23/58 (39.7%)	15/58 (25.9%)	0/59 (0%)
*Neisseria spp (NO ID)*		1/7 (14.2%)	1/7 (14.2%)	0/3 (0.0%)	1/3 (33.3%)	0/3 (0.0%)	3/3 (100%)	0/2 (0%)
*N. gonorrhoeae* (%R)^‡^		17.9	0	0.8	42.7	62.9	4.2	n/a

N^R^, number of resistant isolates; N^T^, total number of isolates tested; n/a, not applicable.

*PEN;* penicillin, *CRO*; ceftriaxone, *CFX*; cefixime, CIP; ciprofloxacin, TET; tetracycline, AZI; azithromycin; GEN; gentamicin.

*Azithromycin based on ECOFF of S < 1 mg/L, ^§^Gentamicin based on previous recommended breakpoint^27^.

^‡^Data from Gonococcal Resistance to Antimicrobials Surveillance Programme, 2020^28^.

The Kruskal-Wallis H was performed only on *N. subflava*, *N. macacae*, *N. perflava* and *N. flavescens* (Table 1). The test demonstrated no statistically significant difference in MIC values between the four *Neisseria* species. (Table 1).

### Genomic analysis and relatedness

Thirty isolates were selected for whole genome sequencing (WGS), covering isolates with ceftriaxone MICs ≥ 0.125 mg/L (15 isolates) and < 0.125 mg/L (four isolates), at least one of each species from the MALDI-ToF identification (six isolates) and three isolates where MALDI-ToF identification was not possible (Supplementary Table S5). The genomic data from the study isolates, along with 61 *Neisseria* reference genomes (Supplementary Table S3), was used to generate cgMLST neighbour joining phylogeny.

**Fig. 3:**
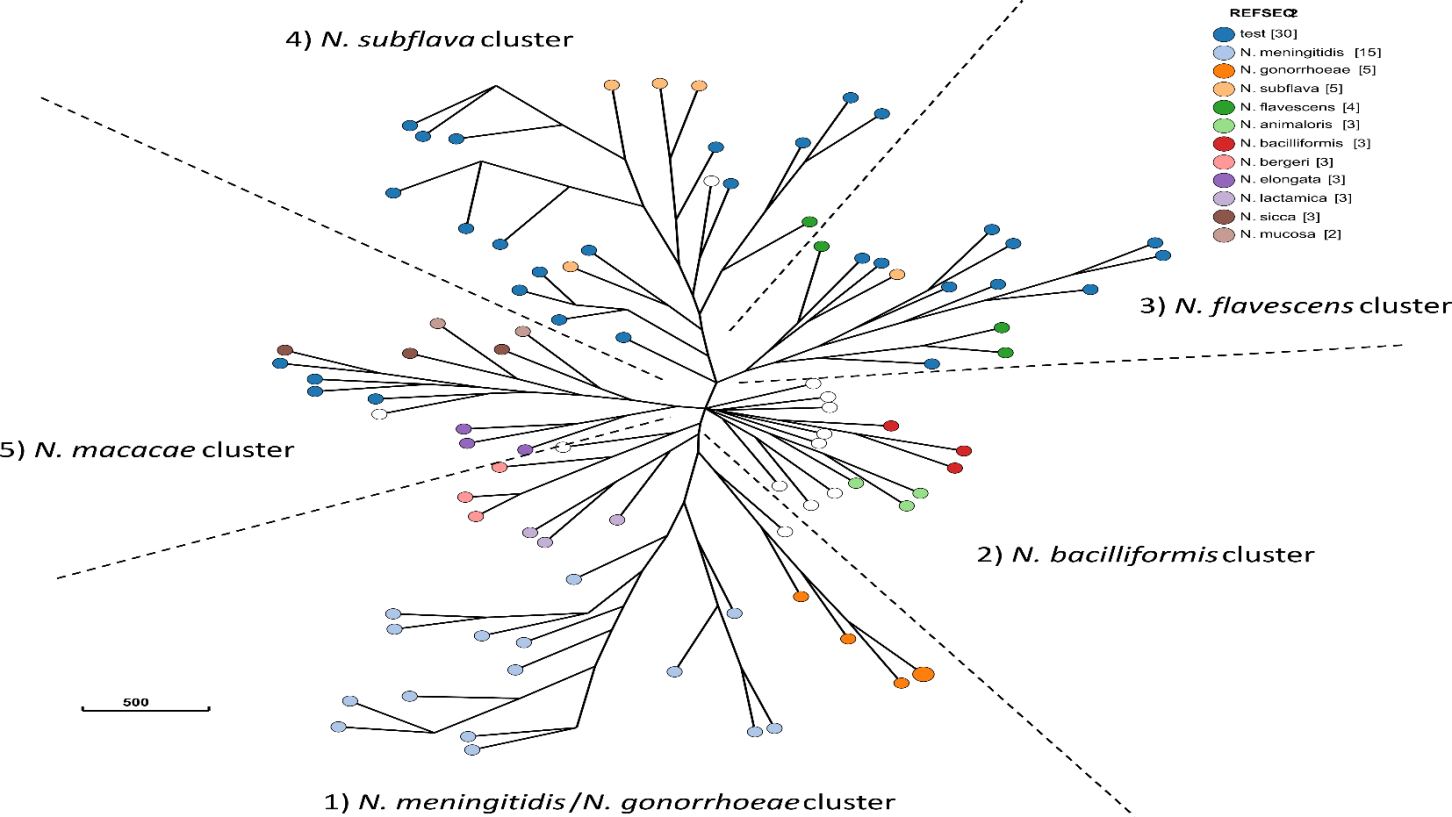
Core genome multi-locus sequence typing (cgMLST) nearest neighbour phylogeny. cgMLST phylogeny derived from 842 gene alleles conserved within 95% of 30 commensal *Neisseria* plus 61 reference *Neisseria* species. Nodes coloured by reference species, study isolates coloured dark blue.

The 91 *Neisseria* isolates clustered in approximately five clusters (Fig. 3). As previously described, *N. meningitidis* and *N. gonorrhoeae* isolates clustered together with *N. lactamica* and *N. polysaccharea*^4^ however *N. bergeri* and *N. cinerea* were also present within the cluster. No study isolates were present in the *N. meningitidis*/*N. gonorrhoeae* cluster (Supplementary Tables S3 and S4). The *N. bacilliformis* group also contained *N. bacilliformis*, *N. animaloris*, and 8 other species but no study isolates (Supplementary Table S3 and S4). MLST analysis of *N. perflava* CCH10-H12, which clustered with *N. mucosa* isolates only matched 3 alleles in the database: *abcZ233*, *adk178* and *pdhC561* (Supplementary Table S3). This combination of alleles was only found together in ST-16,693 but this ST was not associated with any isolates in the database. *abcZ233* was present in ST-3706 (*N. mucosa*), ST-9926 (*N. perflava*), ST-10,150 (*N. mucosa*), ST-16,006 (*N. mucosa*), ST-16,037 (*N. mucosa*), ST-16,480 (*N. mucosa*). *Adk178* was present in ST-3706 (*N. mucosa*) and *pdhC561* was present in ST-12,049 (*N. mucosa*).

The *N. flavescens* cluster contained 3/4 *N. flavescens*, a single *N. subflava* and 10 study isolates. The *N. subflava* cluster contained 4/5 *N. subflava* and 2/2 *N. perflava* plus 16 study isolates. Finally, the *N. macacae* cluster contained 1/1 *N. macacae*, 3/3 *N. elongata*, 3/3 *N. sicca* and 1/1 *N. mucosa* plus four study isolates (Supplementary Tables S3 and S4).

We compared the first and second species identification given by MALDI-ToF and Kraken2 from the genome sequence, excluding the three isolates with no MALDI-ToF ID. A total of 16/26 (61.5%) isolates had ID concordance between the primary MALDI-ToF ID and Kraken2 and 22/26 (84.6%) had concordance between any MALDI-ToF ID and Kraken2 (Supplementary Table S5). The three isolates with no MALDI-ToF ID were predicted as *N. subflava* by Kraken2. All isolates identified as *N. subflava*,* N. perflava* or *N. flavescens* by MALDI-ToF were predicted as *N. subflava* by Kraken2. The isolates identified as *N. macacae* by MALDI-ToF were predicted as *N. mucosa* by Kraken2.

### Genotypic antimicrobial resistance

**Fig. 4:**
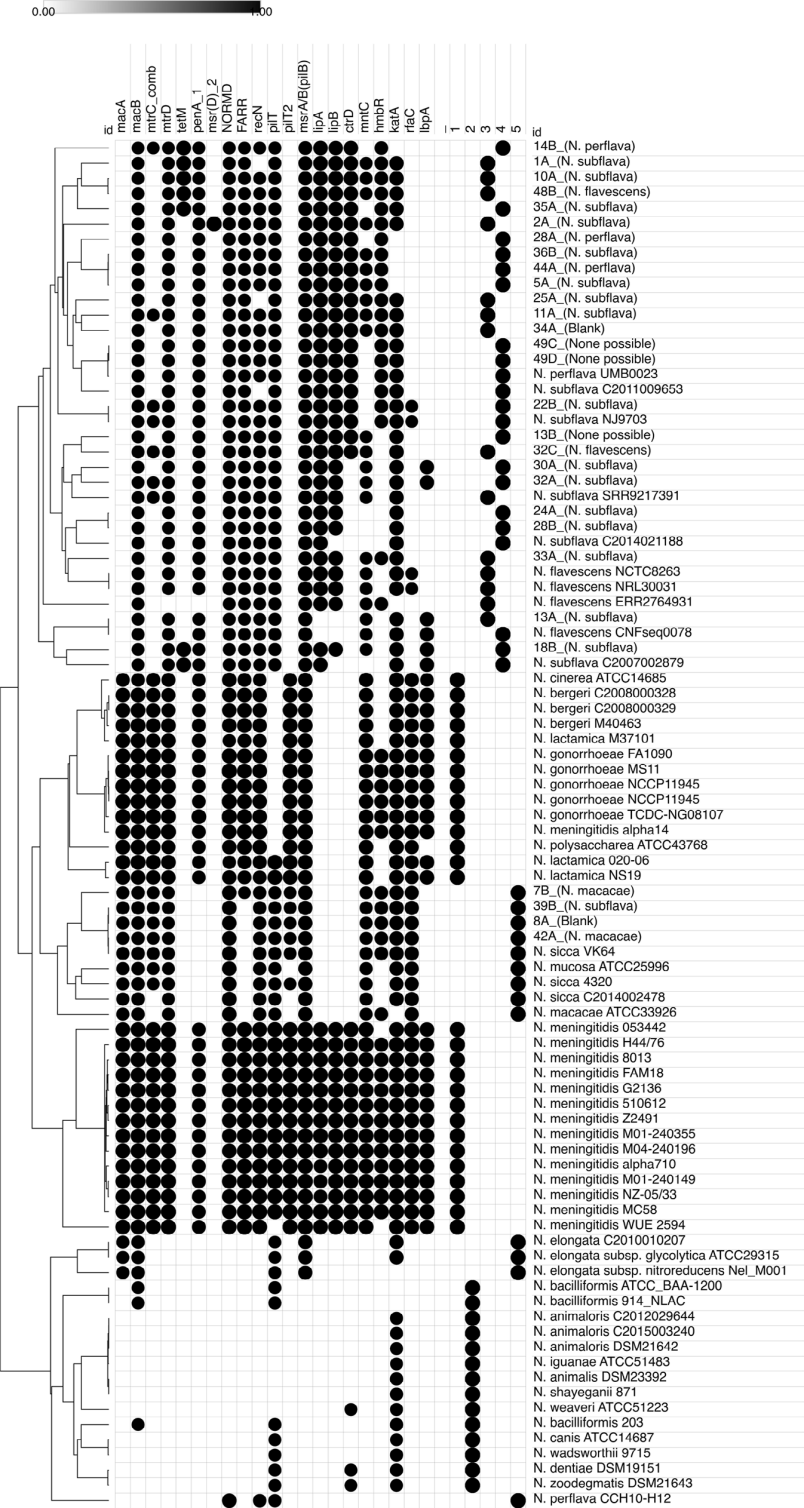
AMR and virulence genes. Draft genomes were analysed for AMR genes (CARD, ResFinder and Megares databases) and virulence (VFDB) genes using Abricate (min ID/coverage 80%). Circles represent the presence of gene, scaled to %ID. Similar profiles were grouped using Euclidean hierarchical clustering using average linkage algorithm in Morpheus. Study isolates are given with MALDI-ToF identification in parenthesis. 1 to 5 indicate cgMLST clustering group; 1 = *N. meningitidis*/*N. gonorrhoeae* cluster, 2 = *N. bacilliformis* cluster, 3 = *N. flavescens* cluster, 4 = *N. subflava* cluster, 5 = *N. macacae* cluster.

One isolate (49 A) produced a poor assembly so was removed from further analysis. Analysis of the remaining 29 *N*c genomes for AMR related genes identified five matches (min 80% identity, 80% coverage) with the CARD database, three with ResFinder, eight with MEGARes additionally 14 virulence related genes with matched against VFDB (Fig. 4).

The MacAB-TolC tripartite macrolide efflux complex consists of *macA*, *macB* and *tolC*. *macB* was present in most isolates except 12/14 of the *N. bacilliformis* cluster isolates and *N. perflava* CCH10-H12, however *macA* was only identified in *N. meningitidis*/*N. gonorrhoeae* cluster isolates and *N. macacae* group isolates plus 49 A^48^ (Fig. 4). Similarly, *mtrC* and *mtrD*, along with *mtrE*, encode a multidrug efflux complex but while *mtrCD* were conserved within *N. meningitidis*/*N. gonorrhoeae* cluster [cluster 1], these genes differentiated the *N. mucosa/sicca/macacae* (present) from *N. elongata* and *N. perflava* CCH10-H12 (absent) within the *N. macacae* cluster^5^. mtrCD was also completely absent from the *N. bacilliformis* cluster^2^. Within the *N. flavescens* and *N. subflava* cluster all isolates except *N. flavescens* ERR2764931 had *mtrD* but only five isolates also had *mtrC*.

PenA, linked to β-lactam resistance, was only present in the *N. meningitidis*/*N. gonorrhoeae*, *N. flavescens* (except *N. flavescens* ERR2764931) and *N. subflava* clusters. TetM, a ribosomal protection protein that confers tetracycline resistance, was present in 7 isolates: *N. subflava* C2007002879, 1 A, 10 A, 14B, 18B, 35 A and 48B, which were spread evenly across *N. flavescens* and *N. subflava* clusters (Fig. 4). Isolates 14B and 18B had tetracycline MICs of 0.5 mg/L and 1 A, 10 A & 35 A had MICs of 16–32 mg/L (Supplementary Table S2). Tetracycline MIC testing was not performed on isolate 48B as it was nonviable on resuscitation.

Capsule polysaccharide modification proteins (LipA/LipB) and capsule polysaccharide export ATP-binding protein (CtrD) were present in all *N. meningitidis* (except *N. meningitidis* alpha14) but absent from *N. gonorrhoeae* and *N. lactamica* Additionally, all three genes were conserved within the majority of *N. flavescens/N. subflava* clusters (*lipA*: 34/36, *lipB*: 32/36, *ctrD*: 22/36) but absent from *N. bergeri*, *N. polysaccharea* and *N. cinerea*.

### Analysis of DNA transfer mechanisms

The *N*c genomes were screened for the presence of gcDUS, AT-DUS and vDUS. All three DUS dialects were found in the *N*c genomes. Overall, the *N. subflava* complex (*N. subflava*,* N. perflava* and *N. flavescens*) isolates had more gcDUS repeats than vDUS whereas the opposite was seen with *N. macaca*e. The *N. subflava* complex isolates had 2738–2990 *Ng* DUS, 144–192 AT-DUS and 158–276 vDUS repeats. *N. macacae* isolates carried 247–292 *Ng* DUS, 29–40 AT-DUS and 3608–3802 vDUS repeats (Supplementary Table S6). No genetic plasmid markers were identified; however, *tetM* has previously been identified as plasmid mediated^49^. Raw reads from all *tetM* positive isolates were mapped against pEP5289 (GU479466, ‘Dutch’ *tetM*) and pEP5050 (GU479464, ‘American’ *tetM* genetic load area) which showed no mapped reads except to the *tetM* gene. Subsequent analysis identified a cryptic 40 kb plasmid in isolate 8 A (*N. macacae*) that had 95% coverage, 99.7% identity to a *Ng* plasmid (CP048906) however this plasmid did not contain any AMR genes.

## Discussion

The value of monitoring carriage and the AMR reservoir of *N*c from the human oropharynx is becoming increasingly evident, not only to prevent the development of AMR in *Nm* and *Ng*, but also the assess the risk of oropharyngeal colonisation and persistence of the pathogenic *Neisseria* species. Not only is there transmission of AMR genes between *Neisseria* species, there is also evidence *N*c are shared between intimate partners^50^, further exacerbating the problem of AMR transmission. In this study we characterised the carriage, genomic relatedness and antimicrobial susceptibility profiles of *N*c species, acquired from the oropharynx of 50 LSHTM volunteers.

In this study, 84% of the study population were colonised with at least one *N*c species. This finding aligns with recent studies reporting *N*c carriage of 68%^21^ and 100%^17^. However, our findings contrasted with those found by Diallo *et al*.^16^ and Le Saux *et al*.^19^ who found a *N*c prevalence of 10.2% and 11.6% respectively. These studies were focused on colonisation of *Nm* and in particular vaccinated individuals, and it has been suggested that both *Nm* and *N*c carriage can be negatively associated with recent meningococcal vaccination^16^. Also, both these studies used Theyer-Martin (TM) media for pathogenic *Neisseria* species, whereas some *N*c species such as *N. cinerea*,* N. subflava* and *N. mucosa* do not grow very well on this media^51^. This was confirmed by the lack of growth of study *N*c on *Ng* selective VCAT agar. LBVT.SNR media, formulated specifically for the isolation of *N*c^18^, aligns with two older studies that used the same media and identified high prevalence of 96.6%^18^ and 100%^20^. Additionally, the study by Sáez *et al*. that found 100% prevalence used both LBVT.SNR and TM media, the latter added specifically to ensure the recovery of *Nm* and *Nl*^20^.

The most common *N*c species found in this study was *N. subflava*, with 61.4% and 74% of participants colonised by this species. The colonisation rate of *N. subflava* is similar found in two recent studies^17,21^, especially when combined with *N. flavescens* and *N. perflava* as previously described^4^. Surprisingly, *N. lactamica* were not isolated from the study participants, however this was likely due to omission of selective media for pathogenic *Neisseria* species. In fact, as part of our quality control checks, a *N. lactamica* laboratory reference strain grew very poorly on SBVT.SNR media. Carriage of *N. lactamica* seems to be variable depending on the population; the prevalence of *N. lactamica* in previous studies ranged from 0.4%^17^ to 17.3%^52^. Interestingly, some studies showed that young children carry *N. lactamica* at much higher rates than adults^16,52^, which could further explain the lack of recover in our study.

Concordance between MALDI-ToF species identification and Kraken2 prediction was just 65.2% when considering the primary species ID. This further demonstrates the challenge of accurate identification in this homogeneous genus, due to the limitations of both technologies. The accuracy of these techniques is only as good as the curation of the database itself demonstrated by several reports of misidentification of *N*c by MALDI-ToF^53–55^. Similarly, genomic identification is limited by the high genetic recombination of *Neisseria* species^6,28,56–58^ coupled with the lack of an internationally accepted genomic identification scheme.

The introduction of more advanced techniques such as WGS, rMLST and cgMLST have led to several re-classifications of existing species and the discovery of novel species^4–6^. In this study, the isolates clustered into three distinct groups, the *N. flavescens*, *N. perflava* and *N. macacae* clusters, in line with previous findings. The clustering agreed with previously suggested re-classifications of *N. perflava* and *N. subflava* into different variants of *N. subflava*^5^. Similarly, it has been suggested that *N. macacae* and *N. mucosa* can be merged into a single *N. mucosa* group^59^, which our cgMLST cluster analysis supports.

Resistance to all antimicrobials except gentamicin and cefixime was high according to both CLSI and EUCAST breakpoints. The median MIC to ceftriaxone was 0.06 mg/L, which although phenotypically susceptible according to both CLSI and EUCAST breakpoints is just 1–2 log_2_ MIC lower than the 0.125–0.25 mg/L breakpoint with one isolate having an MIC of 8 mg/L. This translates to resistance rates of 5% (CLSI) and 13% (EUCAST) compared to *Ng* resistance rates of 0% for the same year in England^28^, but lower than *N*c resistance rates of 28% reported in Vietnam^17^. Differing AMR rates could be due to differences in study populatons, as the study in Vietnam included only men who have sex with men (MSM)^17^. This patient group are described as having a higher likelihood of repeated gonococcal infection and exposure to ceftriaxone, leading to AMR selection pressures on *N*c^17^.

Commensal *Neisseria* species with high ESC MICs pose a significant reservoir for transfer of resistance and development of mosaic genes in pathogenic *Neisseria* species. Although other antimicrobials are no longer used as empirical treatment, resistance to these should not be overlooked, as there has been evidence of macrolide, tetracycline and fluoroquinolone AMR transfer^57^. Investigations of the *Neisseria* resistome have found high resistance to β-lactams, fluoroquinolones encoded by mutations in *gyrA*, tetracylines due to *tetM* as well as TEM-type β-lactamases^60^. Importantly, a recent study demonstrated in vitro transformation of zoliflodacin resistance, a new DNA replication inhibitor evaluated for treatment of *Ng*, from *N*c to *Ng*, suggesting important implications for the introduction of new antimicrobials^61^. In this study, 30 *N*c isolates genomes were analysed for genotypic markers of acquired resistance and we identified several acquired resistance genes. For example, *msr(D)* responsible for high level macrolide resistance (> 256 mg/L)^57^, was present in 2 A which had an MIC of > 256 mg/L. Macrolide resistance has also been associated with overexpression of the MtrCDE efflux pump, which also confers resistance to b-lactams, tetracyclines and fluoroquinolones^62^. The MtrCDE efflux pump is commonly found in *Ng*^62^ and other *Neisseria* species, however correlation between presence of *mtrCDE* and any macrolide resistances was not identified. Similarly, most of our *N*c isolates had *macB*, another efflux pump complex also found in *Ng*^63^, but there was no correlation with phenotypic resistance. Antimicrobial resistance due to overexpression of efflux pumps are associated with specific mutations^64^ and the presence of efflux pumps genes do not necessarily translate to phenotypic resistance.

Transfer of AMR genes between isolates provides a rapid solution to antibiotic treatment compared to accumulation of new genes through evolutionary purposes. Nc are proposed as a possible source of horizontally acquired AMR genes in pathogenic Neisseria, for example horizontal gene transfer of *penA* from *N. lactamica*, *N. macacae*, *N. mucosa* and *N. cinerea* to Ng^58,65–67^. *Neisseria* are naturally competent and therefore naked DNA is a primary method of acquiring new DNA. The Neisseria DUS sequences enhance this DNA uptake. Members of the *N. subflava* and *N. flavescens* clusters had more copies of gcDUS than vDUS and the opposite was true for the *N. macacae* cluster (Supplementary Table S6). These findings agree with previous published data^10,68^ and suggest that DNA incorporation into *Ng* and *Nm* would be more efficient from *N. subflava* and *N. flavescens* clusters than *N. macacae* cluster isolates. Even though *N*c have fewer copies of AT-DUS that enhances transformation efficiency, these findings demonstrate the high likelihood of HGT between *N*c and pathogenic *Neisseria* species, not just relating to AMR, but also virulence and niche adaptation^68^. Plasmids also can transfer AMR genes in Neisseria for example *tetM* was associated with tetracycline resistance in six of our isolates (1 A, 10 A, 14B, 18B, 35 A and 48B), three of which had tetracycline MICs of 16–32 mg/L (1 A, 10 A and 35 A) and two had an MIC of 0.5 mg/L (14B and 18B) (Supplementary Table S2). Tetracycline resistance due to *tetM* is usually coded on a conjugative plasmid in *Ng*, resulting in MICs of 16–64 mg/L^69^. No plasmid markers or known *tetM* carrying plasmids were detected suggesting *tetM* may be present in the chromosome of some *N*c species. Interestingly, a single plasmid was identified in a N. macacae isolate that had previously been sequenced in a Ng isolate. While this supports transfer between pathogenic and commensal Neisseria no AMR genes were present on this plasmid.

In our study we performed comprehensive phenotypic and genotypic analysis of both *N*c carriage, speciation, and AMR determinants, but it is not without limitations. Firstly, our sample size was small, which limited statistical power in some analyses, such as exploring the relationship between *N*c and AMR. Additionally, we did not use *Ng* selective agar, which may have enabled us to recover *N. lactamica* due to the possibility of isolating *Ng/Nm* which was outside the scope of the project and had additional ethical considerations. There is currently no gold standard for speciation of *N*c; the accuracy of genomic and MALDI-ToF analyses are reliant on the accuracy of published reference genomes and identification databases. The nomenclature and speciation of *N*c is evolving, with species reclassified and new species being discovered, meaning that taxonomic errors in reference databases have been discovered^59^. This issue also extends to phenotypic and genotypic analysis of AMR. Firstly, there are no guidelines or resistance breakpoints for *N*c and most published literature have used CLSI or EUCAST breakpoints for *Ng*. This also means there are no international control strains for *N*c susceptibility testing which impacts the accuracy of both phenotypic and genotypic testing. Published fully susceptible *N*c reference genomes will enable detection of single nucleotide polymorphisms and mosaic genes as well as acquired resistances.

This study demonstrated high pharyngeal colonisation rates in our population with higher AMR rates than *Ng*. Although more research in needed to understand the mechanisms of HGT in vivo, monitoring *N*c may help us predict the rates of *Ng* resistant strains occurring in the future, especially relating to ESCs and other newly introduced antimicrobials.

## Electronic supplementary material

Below is the link to the electronic supplementary material.

Supplementary Material 1

## Data Availability

The whole genome datasets presented in this study can be found online at https://www.ebi.ac.uk/ena under study PRJEB67528. Any additional datasets are available from the corresponding author upon reasonable request.
