# Supplementary material for: Carriage and antimicrobial susceptibility of commensal Neisseria species from the human oropharynx
Source: Sci Rep. 2024 Oct 23;14:25017. doi: 10.1038/s41598-024-75130-9 (PMC11499998; doi:10.1038/s41598-024-75130-9)
Supplement: Supplementary file 1 — Supplementary Material 1 [file 41598_2024_75130_MOESM1_ESM.docx]

**Supplementary Files**

**Title:** Carriage and antimicrobial susceptibility of commensal Neisseria species from the human oropharynx

**Authors:** Victoria F Miari^1^*, Wesley Bonnin^1^, Imogen KG Smith^1^, Megan F Horney^1^, Samer J Saint-Geris^2^, Richard A Stabler^1^

^1^Department of Infection Biology, London School of Hygiene & Tropical Medicine, Keppel Street, London, UK

^2^Department of Microbiology, Whittington Health NHS Trust, London, UK

**Supplementary Table S1. Commensal Neisseria species as reported by MALDI-ToF MS, and phenotypic characteristics on LBVT.SN media.** Peritonsillar swabs from 50 participants were spread onto LBVT.SNR agar. Morphologically distinct colonies were sub-cultured onto chocolate agar. Gram stain, oxidase and antimicrobial sensitivity testing were performed on each isolate. Gram-negative, oxidase positive cocci were considered as presumptive *Neisseria spp*. and species determined by MALDI-ToF.

MALDI ID 1; Primary identification (best species match), MALDI ID 2; Secondary identification

MALDI score >1.99; high confidence ID, MALDI score 1.7-1.99; low confidence ID, MALDI score <1.7; No organism ID possible.

ND; none detected, NP; no ID possible, NR; not reported

| Isolate | MALDI ID 1 | MALDI Score 1 | MALDI ID 2 | MALDI Score 2 | Sucrose Fermenter | Colonial Morphology |
| --- | --- | --- | --- | --- | --- | --- |
| 1E | *N. subflava* | 2.05 | *N. subflava* | 2.05 | No | Smooth |
| 1A | *N. subflava* | 2.2 | *N. subflava* | 2.18 | No | Smooth |
| 1B | *N. subflava* | 2.22 | *N. subflava* | 2.19 | Yes | Smooth |
| 2A | *N. subflava* | 1.97 | *N. subflava* | 1.8 | No | Smooth |
| 2B | *N. macacae* | 2.31 | *N. subflava* | 1.91 | Yes | Dry |
| 2C | *Neisseria spp* | ND | NP | NP | No | Smooth |
| 3A | *Neisseria spp* | ND | NP | NP | No | Smooth |
| 3B | *N. subflava* | 2.05 | *N. subflava* | 2.03 | Yes | Smooth |
| 4A | *N. subflava* | 1.89 | *N. subflava* | 1.87 | No | Smooth |
| 4B | *N. subflava* | 2.07 | *N. subflava* | 2.06 | Yes | Dry |
| 5A | *N. subflava* | 2.24 | *N. subflava* | 2.18 | No | Dry |
| 5B | *N. subflava* | 2.21 | *N. subflava* | 2.16 | Yes | Smooth |
| 5C | *N. subflava* | 2.02 | *N. subflava* | 1.98 | Yes | Smooth |
| 5D | *N. flavescens* | 2.05 | *N. subflava* | 2.03 | No | NR |
| 5E | *N. flavescens* | 2.13 | *N. subflava* | 2.1 | Yes | Smooth |
| 7A | *N. subflava* | 1.99 | *N. subflava* | 1.98 | No | Smooth |
| 7B | *N. macacae* | 2.26 | *N. mucosa* | 1.93 | Yes | Smooth |
| 8E | *N. subflava* | 1.91 | *N. flavescens* | 1.88 | No | Smooth |
| 8A | *N. macacae* | 2.14 | *N. subflava* | 1.8 | Yes | Dry |
| 8D | *N. subflava* | 2.31 | *N. subflava* | 2.31 | No | Dry |
| 8F | *N. subflava* | 1.97 | *N. subflava* | 1.9 | NR | Dry |
| 8G | *N. subflava* | 1.91 | *N. flavescens* | 1.89 | NR | NR |
| 8H | *N. macacae* | 2.14 | *N. mucosa* | 1.93 | No | Dry |
| 8I | *N. flavescens* | 2.16 | *N. subflava* | 2.15 | No | Smooth |
| 8J | *Neisseria spp* | ND | None | 1.5 | No | Dry |
| 9A | *N. subflava* | 1.99 | *N. flavescens* | 1.96 | No | Dry |
| 9B | *N. flavescens* | 2.07 | *N. perflava* | 1.82 | No | Smooth |
| 10A | *N. subflava* | 1.94 | *N. subflava* | 1.89 | No | Smooth |
| 10B | *N. subflava* | 2.2 | *N. subflava* | 2.16 | Yes | Smooth |
| 11A | *N. subflava* | 2.13 | *N. subflava* | 1.94 | No | Smooth |
| 11B | *N. subflava* | 2.02 | *N. flavescens* | 2.01 | Yes | Smooth |
| 12A | *N. subflava* | 2.16 | *N. subflava* | 2.11 | No | Smooth |
| 12B | *N. subflava* | 2.12 | *N. subflava* | 2.05 | Yes | Dry |
| 13A | *N. subflava* | 2.08 | *N. subflava* | 2.03 | Yes | Smooth |
| 13B | NP | NP | NP | 1.21 | No | Smooth |
| 14A | *N. flavescens* | 2.04 | *N. perflava* | 2.02 | No | NR |
| 14B | *N. perflava* | 2.17 | *N. flavescens* | 2.03 | No | Smooth |
| 15A | *N. perflava* | 2.04 | *N. subflava* | 2.08 | No | Dry |
| 15B | *N. subflava* | 2.19 | *N. subflava* | 2.08 | No | Smooth |
| 15C | *N. subflava* | 2.1 | *N. subflava* | 2.1 | NR | Dry |
| 18A | NP | NP | NP | NP | No | Smooth |
| 18A | *N. subflava* | 2.11 | *N. perflava* | 2.09 | No | Smooth |
| 19A | *N. mucosa* | 1.92 | *N. cinera* |  | No | NR |
| 20A | *N. subflava* | 2.2 | *N. perflava* | 2.19 | Yes | Smooth |
| 21A | *N. perflava* | 2.15 | *N. flavescens* | 2.1 | Yes | Dry |
| 21B | *N. perflava* | 2.08 | *N. flavescens* | 2.06 | Yes | Smooth |
| 21C | *N. subflava* | 2.15 | *N. subflava* | 2.11 | Yes | Dry |
| 22A | *N. subflava* | 2.15 | *N. subflava* | 2.07 | Yes | Dry |
| 22B | *N. subflava* | 2.03 | *N. flavescens* | 1.96 | No | Smooth |
| 22C | *N. flavescens* | 2.07 | *N. flavescens* | 2.07 | Yes | Smooth |
| 24A | *N. subflava* | 2.36 | *N. subflava* | 2.32 | No | NR |
| 24B | *N. flavescens* | 1.97 | *N. flavescens* | 1.97 | No | Smooth |
| 24C | *N. perflava* | 2.09 | *N. flavescens* | 1.98 | No | Smooth |
| 25A | *N. subflava* | 2.18 | *N. subflava* | 2.12 | No | Smooth |
| 26A | *N. perflava* | 2.03 | *N. subflava* | 1.96 | No | Dry |
| 26B | *N. mucosa* | 2.01 | *N. subflava* | 1.97 | No | Smooth |
| 27A | *N. subflava* | 2.05 | *N. subflava* | 2.01 | No | Dry |
| 28A | *N. perflava* | 2.16 | *N. flavescens* | 2.14 | No | Smooth |
| 28B | *N. subflava* | 2.28 | *N. subflava* | 2.27 | Yes | Smooth |
| 29A | *N. flavescens* | 2.08 | *N. subflava* | 2.08 | Yes | Smooth |
| 30A | *N. subflava* | 2.09 | *N. subflava* | 2.05 | No | Smooth |
| 30B | *N. subflava* | 2.19 | *N. subflava* | 2.18 | Yes | Dry |
| 30C | *N. subflava* | 2.27 | *N. subflava* | 2.18 | Yes | Smooth |
| 32A | *N. subflava* | 2.22 | *N. subflava* | 2.17 | NR | Dry |
| 32B | *N. subflava* | 2.23 | *N. subflava* | 2.22 | No | Smooth |
| 32C | *N. flavescens* | 2.13 | *N. subflava* | 2.12 | Yes | Smooth |
| 33A | *N. subflava* | 2.18 | *N. subflava* | 2.12 | No | Smooth |
| 34A | *N. subflava* | 2.08 | *N. subflava* | 2.03 | Yes | Dry |
| 35B | *N. subflava* | 2.36 | *N. subflava* | 2.3 | Yes | Smooth |
| 35A | *N. subflava* | 2.21 | *N. subflava* | 2.2 | Yes | Smooth |
| 36A | *N. subflava* | 2.15 | *N. subflava* | 2.09 | Yes | Smooth |
| 36B | *N. subflava* | 2.22 | *N. subflava* | 2.15 | No | Smooth |
| 38A | *N. subflava* | 2.01 | *N. subflava* | 1.89 | No | Smooth |
| 38B | *N. subflava* | 1.76 | NP | 1.69 | Yes | Smooth |
| 39A | *N. subflava* | 2.07 | *N. subflava* | 1.99 | No | Smooth |
| 39B | *N. macacae* | 1.81 | *N. mucosa* | 1.75 | Yes | Smooth |
| 40A | *N. flavescens* | 2.01 | *N. subflava* | 1.84 | No | NR |
| 40B | *N. subflava* | 2.16 | *N. subflava* | 2.06 | No | Dry |
| 40C | *N. subflava* | 2.12 | *N. flavescens* | 2.03 | Yes | Smooth |
| 42A | *N. macacae* | 2.15 | *N. mucosa* | 2.02 | Yes | Smooth |
| 43A | *N. subflava* | 2.1 | *N. subflava* | 2.05 | No | Smooth |
| 44E | *Neisseria spp* | ND | NP | ND | No | Smooth |
| 44A | *N. perflava* | 2.14 | *N. subflava* | 2.12 | No | NR |
| 44B | *N. subflava* | 1.95 | *Neisseria spp* | 1.89 | No | Smooth |
| 44C | *N. subflava* | 2.14 | *N. subflava* | 2.14 | No | NR |
| 45A | *N. subflava* | 2.18 | *N. subflava* | 2.17 | Yes | Smooth |
| 45B | *N. subflava* | 2.1 | *N. subflava* | 1.92 | No | Smooth |
| 46A | *N. subflava* | 1.93 | *N. flavescens* | 1.73 | No | Smooth |
| 47A | *N. flavescens* | 2.11 | *N. subflava* | 2 | No | Smooth |
| 47B | *N. flavescens* | 2.18 | *N. subflava* | 2.16 | Yes | NR |
| 48A | *N. subflava* | 2.24 | *N. subflava* | 2.18 | No | Smooth |
| 48B | *N. flavescens* | 1.9 | *N. subflava* | 1.87 | Yes | Smooth |
| 48C | *N. flavescens* | 1.95 | *N. flavescens* | 1.92 | No | NR |
| 49E | *N. subflava* | 2.23 | *N. subflava* | 2.22 | Yes | NR |
| 49A | *N. mucosa* | 2.11 | *N. macacae* | 2.05 | No | Smooth |
| 49C | NP | NP | NP | 1.54 | Yes | Smooth |
| 49D | NP | NP | NP | 1.59 | Yes | Smooth |
| 49F | *N. flavescens* | 1.83 | *N. subflava* | 1.77 | No | Smooth |
| 49B | *N. subflava* | 2.24 | *N. subflava* | 2.18 | Yes | Smooth |
| 50A1 | *Neisseria spp* | ND | NP | 1.33 | No | NR |
| 50B1 | *N. perflava* | 1.73 | *N. flavescens* | 1.72 | NR | Smooth |
| 50C1 | *Neisseria spp* | ND | NP | 1.43 | Yes | NR |

**Supplementary Table S2.** Full MIC data (mg/L) for commensal *Neisseria spp.* Cefixime MIC by gradient strip, all other MICs by agar dilution on gonococcal medium base.

PEN; penicillin, CRO; ceftriaxone, CIP; ciprofloxacin, AZI; azithromycin, GEN; gentamicin, TET; tetracycline, CFX; cefixime,

NV; Not tested as non-viable upon resuscitation.

| Isolate | PEN | CRO | CIP | AZI | GEN | TET | CFX |
| --- | --- | --- | --- | --- | --- | --- | --- |
| 1E | 2 | 0.015 | 0.016 | 4 | 4 | 32 | 0.032 |
| 1A | 0.5 | 0.125 | 1 | 0.125 | 4 | 32 | 0.064 |
| 1B | 0.5 | 0.125 | 0.016 | 0.125 | 4 | 32 | 0.094 |
| 2A | 0.5 | 0.125 | 0.5 | 512 | 4 | 8 | 0.047 |
| 2B | 0.5 | 0.06 | 0.125 | 0.25 | 4 | 2 | 0.047 |
| 2C | 0.03 | NV | NV | NV | NV | NV | NV |
| 3A | 0.25 | 0.125 | 0.5 | 2 | 4 | 0.5 | 0.023 |
| 3B | 0.25 | 0.015 | 0.016 | 0.125 | 2 | 0.25 | 0.38 |
| 4A | 1 | 0.125 | 0.016 | 0.25 | 4 | 1 | 0.094 |
| 4B | 0.5 | 1 | 0.032 | 0.125 | 4 | 4 | 0.064 |
| 5A | 2 | 0.5 | 0.016 | 1 | 4 | 1 | 0.047 |
| 5B | 1 | 0.03 | 0.5 | 1 | 4 | 0.5 | 0.032 |
| 5C | 0.5 | 0.125 | 0.016 | 0.125 | 4 | 0.25 | 0.5 |
| 5D | 0.25 | 0.06 | 0.5 | 4 | 2 | 2 | 0.023 |
| 5E | 0.25 | 0.03 | 0.064 | 0.25 | 4 | 1 | 0.032 |
| 7A | 0.5 | 0.125 | 0.016 | 1 | 4 | 1 | 0.094 |
| 7B | 1 | 0.125 | 0.125 | 2 | 4 | 4 | 0.125 |
| 8E | 4 | 0.015 | 32 | 0.125 | 4 | 1 | 0.064 |
| 8A | 1 | 0.125 | 0.064 | 0.5 | 4 | 0.5 | 0.19 |
| 8D | 2 | 0.015 | 0.032 | 8 | 8 | 0.5 | 0.064 |
| 8F | 4 | 8 | 32 | 0.125 | 4 | 1 | 0.047 |
| 8G | 0.5 | 0.08 | 0.5 | 0.032 | 2 | 0.5 | 0.032 |
| 8H | 4 | 0.06 | 32 | 0.063 | 2 | 0.5 | 0.064 |
| 8I | 2 | 0.25 | 0.016 | 0.032 | 4 | 1 | 0.023 |
| 8J | 0.03 | 0.25 | NV | NV | NV | NV | NV |
| 9A | 1 | 0.06 | 0.016 | 0.125 | 4 | 0.25 | 0.047 |
| 9B | 1 | 0.06 | 0.008 | 0.032 | 2 | 0.25 | 0.064 |
| 10A | 2 | 0.125 | 0.5 | 1.5 | 4 | 32 | 0.125 |
| 10B | 4 | 0.125 | 2 | 0.5 | 4 | 32 | 0.032 |
| 11A | 2 | 0.06 | 0.5 | 1 | 4 | 1 | 0.064 |
| 11B | 1 | 0.06 | 0.016 | 0.125 | 2 | 0.25 | 0.064 |
| 12A | 1 | 0.06 | 0.016 | 0.064 | 4 | 0.25 | 0.023 |
| 12B | 1 | 0.03 | 0.032 | 0.25 | 4 | 0.25 | 0.094 |
| 13A | 4 | 0.06 | 0.5 | 2 | 4 | 1 | 0.023 |
| 13B | 2 | 0.015 | 0.032 | 1.5 | 8 | 1 | 0.094 |
| 14A | 0.5 | 0.25 | 0.032 | 0.25 | 4 | 0.5 | 0.064 |
| 14B | 0.5 | 0.25 | 0.016 | 0.125 | 4 | 0.5 | 0.094 |
| 15A | 2 | 0.06 | 1 | 1 | 2 | 0.5 | 0.047 |
| 15B | 0.5 | 0.06 | 1 | 1 | 4 | 0.5 | 0.032 |
| 15C | 0.25 | 0.125 | 1 | 1 | 4 | 32 | 0.125 |
| 18A | 0.125 | 0.03 | 0.016 | 2 | 4 | 0.25 | 0.047 |
| 18B | 0.125 | 0.008 | 0.008 | 0.75 | 2 | 0.5 | 0.016 |
| 19A | 2 | 0.06 | 0.016 | 1 | 4 | 0.25 | 0.047 |
| 20A | 1 | 0.06 | 0.016 | 4 | 8 | 32 | 0.064 |
| 21A | 0.25 | 4 | 0.5 | 1 | 2 | 0.5 | 0.047 |
| 21B | 0.25 | 0.08 | 0.5 | 1 | 2 | 0.5 | 0.032 |
| 21C | 0.125 | 0.08 | 0.5 | 0.75 | 2 | 0.25 | 0.047 |
| 22A | 1 | 0.06 | 0.008 | 2 | 4 | 0.25 | 0.047 |
| 22B | 0.25 | 0.015 | 0.016 | 0.5 | 4 | 0.25 | 0.032 |
| 22C | 1 | 0.015 | 0.016 | 0.125 | 4 | 0.25 | 0.032 |
| 24A | 0.5 | 0.03 | 2 | 0.06 | 2 | 0.5 | 0.094 |
| 24B | 4 | 0.03 | 0.5 | 0.75 | 4 | 0.5 | 0.125 |
| 24C | 0.5 | 0.06 | 0.5 | 0.75 | 4 | 1 | 0.125 |
| 25A | 0.5 | 0.06 | 0.5 | 1 | 4 | 0.5 | 0.032 |
| 26A | 2 | 0.125 | 0.016 | 4 | 4 | 0.5 | 0.094 |
| 26B | 0.03 | 0.06 | 0.016 | 0.125 | 4 | 0.5 | 0.064 |
| 27A | 2 | 0.06 | 0.5 | 1.5 | 4 | 0.25 | 0.047 |
| 28A | 2 | 0.03 | 0.016 | 1.5 | 4 | 0.125 | 0.064 |
| 28B | 2 | 0.06 | 0.016 | 4 | 4 | 0.5 | 0.094 |
| 29A | 0.125 | 0.08 | 0.016 | 1.5 | 4 | 0.25 | 0.094 |
| 30A | 1 | 0.125 | 0.064 | 2 | 4 | 0.25 | 0.064 |
| 30B | 0.5 | 0.03 | 0.016 | 2 | 4 | 0.25 | 0.094 |
| 30C | 2 | 0.125 | 2 | 512 | 2 | 2 | 0.047 |
| 32A | 2 | 0.06 | 0.032 | 0.063 | 8 | 0.5 | 0.064 |
| 32B | 1 | 0.06 | 0.008 | 0.032 | 4 | 0.25 | 0.064 |
| 32C | 1 | 0.015 | 0.032 | 0.125 | 4 | 0.25 | 0.064 |
| 33A | 0.5 | 0.25 | 8 | 0.032 | 4 | 0.032 | 0.5 |
| 34A | 4 | 0.25 | 0.032 | 0.125 | 4 | 0.5 | 0.125 |
| 35B | 0.125 | 0.03 | 0.032 | 0.032 | 4 | 16 | 0.094 |
| 35A | 0.25 | 0.125 | 0.016 | 0.032 | 4 | 16 | 0.064 |
| 36A | 0.25 | 0.06 | 0.008 | 0.032 | 0.5 | 0.032 | 0.002 |
| 36B | 0.5 | 0.5 | 0.016 | 0.5 | 4 | 0.25 | 0.047 |
| 38A | 0.5 | 0.06 | 0.016 | 0.032 | 4 | 0.25 | 0.047 |
| 38B | 0.5 | 0.06 | 0.5 | 0.032 | 4 | 8 | 0.047 |
| 39A | 0.25 | 0.06 | 0.016 | 0.032 | 8 | 0.25 | 0.032 |
| 39B | 2 | 0.125 | 2 | 2 | 2 | 2 | 0.064 |
| 40A | 1 | 0.06 | 0.5 | 0.032 | 2 | 0.25 | 0.047 |
| 40B | 1 | 0.06 | 0.25 | 0.016 | 2 | 0.25 | 0.094 |
| 40C | 1 | 0.06 | 1 | 0.032 | 2 | 8 | 0.125 |
| 42A | 2 | 0.25 | 1 | 0.5 | 2 | 1 | 0.064 |
| 43A | 1 | 0.06 | 0.008 | 0.016 | 4 | 0.5 | 0.094 |
| 44A | 1 | 0.06 | NV | NV | NV | NV | NV |
| 44E | 2 | 0.06 | 0.016 | 0.016 | 4 | 0.5 | 0.125 |
| 44A | 0.5 | 0.06 | 0.016 | 0.032 | 4 | 0.25 | 0.032 |
| 44B | 2 | 0.06 | 0.008 | 0.016 | 4 | 0.5 | 0.094 |
| 45A | 1 | 0.06 | 0.016 | 1.5 | 4 | 4 | 0.047 |
| 45B | 0.5 | 0.03 | 0.032 | 3 | 4 | 0.25 | 0.047 |
| 46A | 0.5 | 0.125 | 0.008 | 1 | 2 | 16 | 0.125 |
| 47A | 1 | 0.06 | 0.016 | 1.5 | 4 | 0.25 | 0.032 |
| 47B | 1 | 0.25 | 2 | 4 | 16 | 1 | 0.5 |
| 48A | 1 | 0.125 | NV | NV | NV | NV | NV |
| 48B | 4 | 0.125 | NV | NV | NV | NV | NV |
| 48C | 1 | 0.015 | NV | NV | NV | NV | NV |
| 49E | 1 | 0.06 | 0.5 | 0.032 | 2 | 1 | 0.064 |
| 49A | 0.5 | 0.06 | 0.25 | 0.032 | 2 | 0.5 | 0.047 |
| 49C | 0.03 | 0.015 | 0.5 | 1 | 2 | 0.5 | 0.064 |
| 49F | 1 | 0.06 | 0.5 | 0.032 | 2 | 1 | 0.047 |
| 49B | 1 | 0.015 | 0.016 | 3 | 4 | 0.5 | 0.023 |
| 50A | 0.5 | 0.06 | NV | NV | NV | NV | NV |
| 50B | 0.125 | 0.015 | NV | NV | NV | NV | NV |
| 50C | 0.03 | 0.015 | NV | NV | NV | NV | NV |

**Supplementary Table 3 Reference genomes.** High quality reference genomes used in this study. Multilocus sequence type (MLST) designated sequence type (ST) is derived from unique combination of *abcZ, adk, aroE, fumC, gdh, pdhC, pgm* alleles. ~n denotes a novel full-length allele with ≥ 95% identity to allele number ‘n’. n? denotes a partial match to known allele with ≥ 10% coverage and identity ≥ 95%. – denotes no match to existing alleles with ≥ 10% coverage and identity ≥ 95%. Reference and study isolates were used to generate a whole genome MLST (wgMLST) schema and a nearest neighbour phylogeny consisting of 5 main clusters labelled; 1. Nm/Ng, 2. *N. bacilliformis*, 3. *N. flavescens*, 4. *N. subflava*, 5. *N. macacae*.

| **Accession ID** | **Species** | **Strain** | **ST** | **abcZ** | **adk** | **aroE** | **fumC** | **gdh** | **pdhC** | **pgm** | **wgMLST group** |
| --- | --- | --- | --- | --- | --- | --- | --- | --- | --- | --- | --- |
| **RKRJ01** | *N. animalis* | *N. animalis DSM 23392* | - | 528 | 357 | 621 | - | 551 | 552 | 551 | *N. bacilliformis* |
| **MTBN01** | *N. animaloris* | *N. animaloris DSM 21642* | - | - | - | - | - | - | - | - | *N. bacilliformis* |
| **POXR01** | *N. animaloris* | *N. animaloris C2012029644* | - | - | - | - | - | - | 771 | - | *N. bacilliformis* |
| **POYC01** | *N. animaloris* | *N. animaloris C2015003240* | - | - | - | - | - | - | - | - | *N. bacilliformis* |
| **AFAY01** | *N. bacilliformis* | *N. bacilliformis* ATCC BAA-1200 | 9330 | 552 | 353 | 605 | 529 | 589 | 548 | 545 | *N. bacilliformis* |
| **JUOC01** | *N. bacilliformis* | *N. bacilliformis 914_NLAC* | - | 552? | 374 | 605? | 553 | ~589 | ~586 | ~568 | *N. bacilliformis* |
| **JVQC01** | *N. bacilliformis* | *N. bacilliformis* 203 | - | 552? | ~359 | ~664 | ~525 | ~638 | ~586 | 641 | *N. bacilliformis* |
| **MTBL01** | *N. canis* | *N. canis ATCC 14687* | - | 540 | 377 | 625 | - | 574 | 571 | 570 | *N. bacilliformis* |
| **MTBO01** | *N. dentiae* | *N. dentiae DSM 19151* | 9335 | 539 | 376 | 624 | 554 | 572 | 570 | 569 | *N. bacilliformis* |
| **PXYY01** | *N. iguanae* | *N. iguanae ATCC 51483* | - | - | - | - | - | - | - | - | *N. bacilliformis* |
| **AGAY01** | *N. shayeganii* | *N. shayeganii* 871 | - | 596 | 417 | - | - | - | 656 | 656 | *N. bacilliformis* |
| **AGAZ01** | *N. wadsworthii* | N. wadsworthii 9715 | - | 595 | 416 | 669 | - | 643 | 623 | 655 | *N. bacilliformis* |
| **AFWR01** | *N. weaveri* | *N. weaveri* ATCC 51223 | - | 529 | 358 | 618 | 533 | 587 | ~553 | 552? | *N. bacilliformis* |
| **MTBM01** | *N. zoodegmatis* | N. zoodegmatis DSM 21643 | - | ~746 | ~516 | 791? | ~752 | 757? | ~769 | 552? | *N. bacilliformis* |
| **ACEN01** | *N. flavescens* | *N. flavescens* NRL30031 | 3576 | 244 | 172 | 296 | 285 | 270 | 259 | 276 | *N. flavescens* |
| **UGQV01** | *N. flavescens* | *N. flavescens NCTC8263* | 3576 | 244 | 172 | 296 | 285 | 270 | 259 | 276 | *N. flavescens* |
| **CAJZIH01** | *N. flavescens* | *N. flavescens* ERR2764931_  bin.5_metaWRAP_v1.1_MAG | - | ~822 | - | ~307 | ~879 | 272 | ~573 | ~270 | *N. flavescens* |
| **LAEK01** | *N. flavescens* | *N. flavescens CNF seq0078* | - | ~271 | 538? | ~511 | ~854 | ~276 | ~792 | ~270 | *N. flavescens* |
| **CAJPLX01** | *N. subflava* | *N. subflava* SRR9217391-mag-bin.22 | - | 527? | ~171 | ~883 | 86? | ~566 | ~512 | ~502 | *N. flavescens* |
| **ADBF01** | *N. elongata* | *N. elongata* subsp. *glycolytica* ATCC 29315 | 9806 | 493 | 330 | 604 | 491 | 584 | 542 | 540 | *N. macacae* |
| **JAGJWT01** | *N. elongata* | *N. elongata subsp. nitroreducens Nel_M001* | - | ~776 | ~328 | ~604 | ~692 | ~588 | ~534 | ~540 | *N. macacae* |
| **POXH01** | *N. elongata* | *N. elongata C2010010207* | - | ~312 | ~328 | ~607 | ~487 | ~586 | ~550 | ~546 | *N. macacae* |
| **AFQE01** | *N. macacae* | *N. macacae* ATCC 33926 | 9339 | 525 | 354 | 577 | 530 | 548 | 549 | 547 | *N. macacae* |
| **ACDX02** | *N. mucosa* | *N. mucosa* ATCC 25996 | 8082 | 492 | 329 | 546 | 490 | 520 | 513 | 503 | *N. macacae* |
| **AEPF01** | *N. sicca* | *N. sicca* 4320 | 3707 | 236 | 170 | 317 | 268 | 274 | 288 | 264 | *N. macacae* |
| **AJMT01** | *N. sicca* | *N. sicca* VK64 | 10254 | 526 | 355 | 578 | 531 | 549 | 613 | 548 | *N. macacae* |
| **POXX01** | *N. sicca* | *N. sicca C2014002478* | - | ~236 | ~367 | 583 | ~743 | ~520 | ~804 | ~547 | *N. macacae* |
| **LSIT01** | *N. perflava* | *N. perflava CCH10-H12* | - | 233 | 178 | - | - | - | 561 | - | *N. subflava* |
| **PKJQ01** | *N. perflava* | *N. perflava UMB0023* | - | 770 | 543 | 820 | ~299 | 785 | 794 | 810 | *N. subflava* |
| **ACEO02** | *N. subflava* | *N. subflava* NJ9703 | 9805 | 490 | 345 | 306 | 488 | 269 | 277 | 505 | *N. subflava* |
| **POWV01** | *N. subflava* | *N. subflava C2007002879* | - | ~19 | ~538 | ~244 | 895 | ~269 | ~308 | ~898 | *N. subflava* |
| **POXL01** | *N. subflava* | *N. subflava C2011009653* | - | 685? | ~578 | ~883 | 296? | ~823 | ~568 | 844 | *N. subflava* |
| **POYB01** | *N. subflava* | *N. subflava C2014021188* | - | ~836 | ~542 | ~883 | ~487 | ~272 | ~308 | ~272 | *N. subflava* |
| **POWY01** | *N. bergeri* | *N. bergeri C2008000328* | 3558 | 225 | 166 | 23 | 255 | 278 | 269 | 271 | *Nm/Ng* |
| **POWZ01** | *N. bergeri* | *N. bergeri C2008000329* | 3558 | 225 | 166 | 23 | 255 | 278 | 269 | 271 | *Nm/Ng* |
| **QQHX01** | *N. bergeri* | *N. bergeri M40463* | 12190 | 495 | 257 | 23 | 29 | 538 | 28 | 529 | *Nm/Ng* |
| **ACDY02** | *N. cinerea* | *N. cinerea* ATCC 14685 | 3579 | 247 | 167 | 284 | 298 | 283 | 284 | 274 | *Nm/Ng* |
| **AE004969** | *N. gonorrhoeae* | *N. gonorrhoeae* FA 1090 | 1899 | 109 | 39 | 67 | 190 | 147 | 71 | 65 | *Nm/Ng* |
| **CP001050** | *N. gonorrhoeae* | *N. gonorrhoeae* NCCP11945 | 1901 | 109 | 39 | 170 | 111 | 148 | 153 | 65 | *Nm/Ng* |
| **NC_011035** | *N. gonorrhoeae* | *N. gonorrhoeae* NCCP11945 | 1901 | 109 | 39 | 170 | 111 | 148 | 153 | 65 | *Nm/Ng* |
| **CP003909** | *N. gonorrhoeae* | *N. gonorrhoeae* MS11 | 6959 | 126 | 39 | 67 | 78 | 146 | 153 | 133 | *Nm/Ng* |
| **CP002440** | *N. gonorrhoeae* | *N. gonorrhoeae* TCDC-NG08107 | 7363 | 59 | 39 | 67 | 78 | 148 | 153 | 65 | *Nm/Ng* |
| **FN995097** | *N. lactamica* | *N. lactamica* 020-06 | 640 | 84 | 49 | 48 | 50 | 92 | 46 | 45 | *Nm/Ng* |
| **AEPI01** | *N. lactamica* | *N. lactamica* NS19 | 12442 | 61 | 511 | 328 | 66 | 625 | 218 | 196 | *Nm/Ng* |
| **QQLL01** | *N. lactamica* | *N. lactamica M37101* | - | 83 | 37 | 87 | 45 | 90 | 44 | ~168 | *Nm/Ng* |
| **AL157959** | *N. meningitidis* | *N. meningitidis serogroup A Z2491* | 4 | 1 | 3 | 3 | 1 | 4 | 2 | 3 | *Nm/Ng* |
| **FR774048** | *N. meningitidis* | *N. meningitidis* WUE 2594 | 5 | 1 | 1 | 2 | 1 | 3 | 2 | 3 | *Nm/Ng* |
| **CP007524** | *N. meningitidis* | *N. meningitidis 510612* | 7 | 1 | 1 | 2 | 1 | 3 | 2 | 19 | *Nm/Ng* |
| **CP002419** | *N. meningitidis* | *N. meningitidis* G2136 | 8 | 2 | 3 | 7 | 2 | 8 | 5 | 2 | *Nm/Ng* |
| **AM421808** | *N. meningitidis* | *N. meningitidis* serogroup C FAM18 | 11 | 2 | 3 | 4 | 3 | 8 | 4 | 6 | *Nm/Ng* |
| **CP002420** | *N. meningitidis* | *N. meningitidis* H44/76 | 32 | 4 | 10 | 5 | 4 | 6 | 3 | 8 | *Nm/Ng* |
| **CP002421** | *N. meningitidis* | *N. meningitidis* M01-240149 | 41 | 3 | 6 | 9 | 5 | 9 | 6 | 9 | *Nm/Ng* |
| **CP002424** | *N. meningitidis* | *N. meningitidis* NZ-05/33 | 42 | 10 | 6 | 9 | 5 | 9 | 6 | 9 | *Nm/Ng* |
| **AM889136** | *N. meningitidis* | *N. meningitidis* alpha14 | 53 | 16 | 2 | 6 | 25 | 17 | 25 | 22 | *Nm/Ng* |
| **AE002098** | *N. meningitidis* | *N. meningitidis* MC58 | 74 | 4 | 10 | 5 | 4 | 5 | 3 | 2 | *Nm/Ng* |
| **CP001561** | *N. meningitidis* | *N. meningitidis* alpha710 | 136 | 27 | 6 | 9 | 3 | 9 | 6 | 16 | *Nm/Ng* |
| **FM999788** | *N. meningitidis* | *N. meningitidis* 8013 | 177 | 7 | 8 | 10 | 38 | 10 | 1 | 20 | *Nm/Ng* |
| **CP002422** | *N. meningitidis* | N. meningitidis M01-240355 | 213 | 7 | 5 | 1 | 13 | 36 | 53 | 15 | *Nm/Ng* |
| **CP002423** | *N. meningitidis* | *N. meningitidis* M04-240196 | 269 | 4 | 10 | 15 | 9 | 8 | 11 | 9 | *Nm/Ng* |
| **CP000381** | *N. meningitidis* | *N. meningitidis* 053442 | 4821 | 222 | 3 | 58 | 275 | 30 | 5 | 255 | *Nm/Ng* |
| **ADBE01** | *N. polysaccharea* | *N. polysaccharea* ATCC 43768 | 3557 | 106 | 66 | 40 | 46 | 219 | 43 | 261 | *Nm/Ng* |

**Supplementary Table S4. Commensal genomes.**

Multilocus sequence type (MLST) designated sequence type (ST) is derived from unique combination of abcZ, adk, aroE, fumC, gdh, pdhC, pgm alleles. ~n denotes a novel full-length allele with ≥ 95% identity to allele number ‘n’. n? denotes a partial match to known allele with ≥ 10% coverage and identity ≥ 95%. – denotes no match to existing alleles with ≥ 10% coverage and identity ≥ 95%. PubMLST denotes Neisseria species associated with the given MLST ST in the PubMLST database (accessed Aug 2022). Match6 denotes Neisseria species that share 6 out 7 alleles in the PubMLST database. Match5 denotes Neisseria species that share 5 out 7 alleles. Match4 denotes Neisseria species that share 4 out 7 alleles. MALDI-ToF was the species predicted by matrix-assisted laser desorption/ionization time of flight analysis.

Reference and study isolates were used to generate a whole genome MLST (wgMLST) schema and a nearest neighbour phylogeny consisting of 5 main clusters labelled; 1. Nm/Ng, 2. *N. bacilliformis*, 3. *N. flavescens*, 4. *N. subflava*, 5. *N. macacae*.

NOP; No organism possible

| Isolate | ST | abcZ | adk | aroE | fumC | gdh | pdhC | pgm | PubMLST | Match6 | Match5 | Match4 | MALDI Primary ID | wgMLST group |
| --- | --- | --- | --- | --- | --- | --- | --- | --- | --- | --- | --- | --- | --- | --- |
| 10A | - | ~57 | ~453 | - | ~634 | ~823 | ~800 | ~555 |  |  |  |  | *N. subflava* | *N. flavescens* |
| 11A | - | 495? | ~382 | ~883 | 612? | 303? | ~308 | ~555 |  |  |  |  | *N. subflava* | *N. flavescens* |
| 13A | - | 536 | ~431 | ~581 | ~269 | ~695 | ~512 | ~557 |  |  |  |  | *N. subflava* | *N. flavescens* |
| 13B | - | ~822 | ~159 | 581? | ~905 | ~350 | 790 | ~566 |  |  |  |  | *NOP* | *N. subflava* |
| 14B | - | 495? | 180 | ~848 | 488 | 269 | 277 | 260 |  |  |  | *N subflava* | *N. perflava* | *N. subflava* |
| 18A | - | 685 | 453 | 738 | 488? | ~707 | 308 | ~809 |  |  |  |  | *N. subflava* | *N. subflava* |
| 1A | - | ~57 | ~382 | ~883 | ~777 | 303? | ~800 | ~393 |  |  |  |  | *N. subflava* | *N. flavescens* |
| 22B | - | ~537 | ~615 | ~441 | ~488 | ~269 | ~568 | ~267 |  |  |  |  | *N. subflava* | *N. subflava* |
| 24A | - | ~237 | ~174 | ~816 | ~363 | ~850 | ~551 | ~283 |  |  |  |  | *N. subflava* | *N. subflava* |
| 25A | - | 770 | ~382 | ~883 | ~777 | 303? | ~800 | ~393 |  |  |  |  | *N. subflava* | *N. flavescens* |
| 28A | - | 231 | 180 | ~848 | ~296 | 269 | 277 | 260 |  |  | *N. subflava* |  | *N. perflava* | *N. subflava* |
| 28B | - | ~382 | 538 | ~816 | 286? | ~545 | 800 | ~898 |  |  |  |  | *N. subflava* | *N. subflava* |
| 2A | - | 836 | ~393 | ~883 | 895 | 276? | 857 | ~554 |  |  |  |  | *N. subflava* | *N. flavescens* |
| 30A | - | 685 | 453 | 738 | 698 | 707 | 711 | 566? |  | *N. subflava* |  |  | *N. subflava* | *N. subflava* |
| 32A | - | 685 | 453 | 738 | 698 | 707 | 711 | 893? |  | *N. subflava* |  |  | *N. subflava* | *N. subflava* |
| 32C | - | ~233 | ~225 | ~813 | ~269 | ~545 | ~666 | ~797 |  |  |  |  | *N. flavescens* | *N. flavescens* |
| 33A | - | 785 | ~597 | ~587 | 794 | ~545 | 800 | 822? |  |  |  |  | *N. subflava* | *N. flavescens* |
| 34A | - | ~267 | ~431 | ~883 | ~488 | 303? | ~800 | ~283 |  |  |  |  | *N. subflava* | *N. flavescens* |
| 35A | - | ~386 | 180 | ~307 | ~488 | 269 | 277 | ~505 |  |  |  |  | *N. subflava* | *N. subflava* |
| 36B | - | ~783 | ~180 | ~883 | 286? | 269 | 277 | 260 |  |  |  |  | *N. subflava* | *N. subflava* |
| 39B | - | ~591 | ~354 | ~585 | ~86 | ~520 | ~561 | ~547 |  |  |  |  | *N. macacae* | *N. macacae* |
| 42A | - | ~236 | ~438 | 311? | ~692 | ~267 | ~707 | ~262 |  |  |  |  | *N. macacae* | *N. macacae* |
| 44A | - | 231 | 180 | 306 | ~488 | 269 | 277 | 260 |  | *N. subflava* |  |  | *N. perflava* | *N. subflava* |
| 48B | 12596 | 785 | 554 | 833 | 794 | 798 | 800 | 822 | *N. subflava* |  |  |  | *N. flavescens* | *N. flavescens* |
| 49A | - | ~19 | 174? | ~848 | 86? | ~275 | ~793 | ~502 |  |  |  |  | *N. mucosa* | *N. subflava* |
| 49C | - | ~19 | 174? | ~848 | 86? | ~275 | ~793 | ~502 |  |  |  |  | *NOP* | *N. subflava* |
| 49D | - | ~19 | 174? | ~848 | 86? | ~275 | ~793 | ~502 |  |  |  |  | *NOP* | *N. subflava* |
| 5A | - | 762 | 180 | 306 | 488 | 778 | 277 | 260 |  | *N. subflava* |  |  | *N. subflava* | *N. subflava* |
| 7B | - | ~638 | 438 | ~311 | ~620 | ~701 | 707 | 642? |  |  |  |  | *N. macacae* | *N. macacae* |
| 8A | - | 526 | ~355 | 578 | ~696 | 549 | 613 | ~691 |  |  |  | *N. mucosa* | *N. macacae* | *N. macacae* |

**Supplementary Table S5:** Agreement of MALDI-ToF with Kraken2 identifications. MALDI ID 1; Primary identification (best species match), MALDI ID 2; Secondary identification.

MALDI score >1.99; high confidence ID, MALDI score 1.7-1.99; low confidence ID, MALDI score <1.7; No organism ID possible.

N/A; Not applicable

*Poor genome assembly

| **Isolate** | **MALDI-ToF ID 1** | **MALDI-ToF 1 SCORE** | **MALDI-ToF ID 2** | **MALDI-ToF 2 SCORE** | **Kraken2 ID** | **Agreement with MALDI-ToF ID 1** | **Agreement with at least 1 MALDI-ToF ID** | **Reason for sequencing** |
| --- | --- | --- | --- | --- | --- | --- | --- | --- |
| 1A | *N. subflava* | 2.2 | *N. subflava* | 2.18 | *N. subflava* | Y | Y | CRO MIC ≥0.125 |
| 2A | *N. subflava* | 1.97 | *N. subflava* | 1.8 | *N. subflava* | Y | Y | CRO MIC ≥0.125 |
| 5A | *N. subflava* | 2.24 | *N. subflava* | 2.18 | *N. subflava* | Y | Y | CRO MIC ≥0.125 |
| 7B | *N. macacae* | 2.26 | *N. mucosa* | 1.93 | *N. mucosa* | N | Y | CRO MIC ≥0.125 |
| 8A | *N. macacae* | 2.14 | *N. subflava* | 1.8 | *N. mucosa* | N | N | CRO MIC ≥0.125 |
| 10A | *N. subflava* | 1.94 | *N. subflava* | 1.89 | *N. subflava* | Y | Y | CRO MIC ≥0.125 |
| 11A | *N. subflava* | 2.13 | *N. subflava* | 1.94 | *N. subflava* | Y | Y | Representative of *N. subflava* |
| 13A | *N. subflava* | 2.08 | *N. subflava* | 2.03 | *N. subflava* | Y | Y | Participant with >1 isolates |
| 13B | *No organism possible* | 1.23 | *No organism possible* | 1.21 | *N. subflava* | N/A | N/A | No ID by MALDI-ToF |
| 14B | *N. perflava* | 2.17 | *N. flavescens* | 2.03 | *N. subflava* | N | N | CRO MIC ≥0.125 |
| 18A | *N. subflava* | 2.11 | *N. perflava* | 2.09 | *N. subflava* | Y | Y | CRO MIC <0.125 |
| 22B | *N. subflava* | 2.03 | *N. flavescens* | 1.96 | *N. sublfava* | Y | Y | CRO MIC ≥0.125 |
| 24A | *N. subflava* | 2.36 | *N. subflava* | 2.32 | *N. subflava* | Y | Y | Representative of *N. subflava* |
| 25A | *N. subflava* | 2.18 | *N. subflava* | 2.12 | *N. subflava* | Y | Y | CRO MIC <0.125 |
| 28A | *N. perflava* | 2.16 | *N. flavescens* | 2.14 | *N. subflava* | N | N | Representative of *N. perflava* |
| 28B | *N. subflava* | 2.28 | *N. subflava* | 2.27 | *N. subflava* | Y | Y | CRO MIC <0.125 |
| 30A | *N. subflava* | 2.09 | *N. subflava* | 2.05 | *N. subflava* | Y | Y | CRO MIC ≥0.125 |
| 32A | *N. subflava* | 2.22 | *N. subflava* | 2.17 | *N. subflava* | Y | Y | Participant with >1 isolates |
| 32C | *N. flavescens* | 2.13 | *N. subflava* | 2.12 | *N. subflava* | N | N | Representative for *N. flavescens* |
| 33A | *N. subflava* | 2.18 | *N. subflava* | 2.12 | *N. subflava* | Y | Y | CRO MIC ≥0.125 |
| 34A | *N. subflava* | 2.08 | *N. subflava* | 2.03 | *N. subflava* | Y | Y | CRO MIC ≥0.125 |
| 35A | *N. subflava* | 2.21 | *N. subflava* | 2.2 | *N. subflava* | Y | Y | CRO MIC ≥0.125 |
| 36B | *N. subflava* | 2.22 | *N. subflava* | 2.15 | *N. subflava* | Y | Y | CRO MIC ≥0.125 |
| 39B | *N. macacae* | 1.81 | *N. mucosa* | 1.75 | *N. mucosa* | N | Y | CRO MIC ≥0.125 |
| 42A | *N. macacae* | 2.15 | *N. mucosa* | 2.02 | *N. mucosa* | N | Y | Representative of *N. macacae* |
| 44A | *N. perflava* | 2.14 | *N. subflava* | 2.12 | *N. subflava* | N | Y | CRO MIC <0.125 |
| 48B | *N. flavescens* | 1.9 | *N. subflava* | 1.87 | *N. subflava* | N | Y | CRO MIC ≥0.125 |
| 49A | *N. mucosa* | 2.11 | *N. macacae* | 2.05 | *None** | Y | Y | Representative of *N. mucosa* |
| 49C | *No organism possible* | 1.6 | *None* | 1.54 | *N. subflava* | n/a | N/A | No ID by MALDI-ToF |
| 49D | *No organism possible* | 1.62 | *No organism possible* | 1.59 | *N. subflava* | N/A | N/A | No ID by MALDI-ToF |

**Supplementary Table S6.** DNA Uptake Sequence Information

***AT-DUS****; Neisseria gonorrhoeae 12 base pair DNA uptake sequence - 5’-AT-GCCGTCTGAA-3’*

***vDUS****; Commensal Neisseria variant DNA uptake sequence (vDUS) – 5’-GTCGTCTGAA-3’*

***DUS****; Neisseria gonorrhoeae 10 base pair DNA uptake sequence (DUS) - 5’-GCCGTCTGAA-3’*

| Isolate | Species by MALDI | Species by Kraken2 | AT-DUS | vDUS | DUS |
| --- | --- | --- | --- | --- | --- |
| 10A | *N. subflava* | *N. subflava* | 165 | 203 | 2766 |
| 11A | *N. subflava* | *N. subflava* | 168 | 169 | 2754 |
| 13A | *N. subflava* | *N. subflava* | 174 | 276 | 2641 |
| 13B | *No ID* | *N. subflava* | 173 | 176 | 2717 |
| 14B | *N. perflava* | *N. subflava* | 168 | 167 | 2822 |
| 18B | *N. subflava* | *N. subflava* | 177 | 209 | 2749 |
| 1A | *N. subflava* | *N. subflava* | 164 | 179 | 2740 |
| 22B | *N. subflava* | *N. subflava* | 159 | 165 | 2759 |
| 24A | *N. subflava* | *N. subflava* | 152 | 183 | 2720 |
| 25A | *N. subflava* | *N. subflava* | 161 | 173 | 2733 |
| 28A | *N. perflava* | *N. subflava* | 169 | 181 | 2761 |
| 28B | *N. subflava* | *N. subflava* | 144 | 217 | 2727 |
| 2A | *N. subflava* | *N. subflava* | 192 | 158 | 2766 |
| 30A | *N. subflava* | *N. subflava* | 177 | 194 | 2620 |
| 32A | *N. subflava* | *N. subflava* | 184 | 218 | 2737 |
| 32C | *N. flavescens* | *N. subflava* | 178 | 198 | 2560 |
| 33A | *N. subflava* | *N. subflava* | 167 | 205 | 2774 |
| 34A | *N. subflava* | *N. subflava* | 158 | 185 | 2705 |
| 35A | *N. subflava* | *N. subflava* | 157 | 181 | 2691 |
| 36B | *N. subflava* | *N. subflava* | 158 | 173 | 2753 |
| 39B | *N. macacae* | *N. mucosa* | 32 | 3801 | 250 |
| 42A | *N. macacae* | *N. mucosa* | 29 | 3795 | 220 |
| 44A | *N. perflava* | *N. subflava* | 172 | 165 | 2741 |
| 48B | *N. flavescens* | *N. subflava* | 165 | 180 | 2785 |
| 49C | *No ID* | *N. subflava* | 165 | 228 | 2758 |
| 49D | *No ID* | *N. subflava* | 170 | 235 | 2796 |
| 5A | *N. subflava* | *N. subflava* | 165 | 173 | 2696 |
| 7B | *N. macacae* | *N. macacae* | 39 | 3608 | 208 |
| 8A | *N. macacae* | *N. mucosa* | 35 | 3802 | 257 |
